# COVID-19 clusters in Malaysia: characteristics, detection methods and modes of early transmission

**DOI:** 10.5365/wpsar.2023.14.4.1058

**Published:** 2023-11-29

**Authors:** Zen Yang Ang, Nur Zahirah Balqis-Ali, Anis-Syakira Jailani, Yuke-Lin Kong, Shakirah Md Sharif, Weng Hong Fun

**Affiliations:** a Institute for Health Systems Research, National Institutes of Health, Ministry of Health, Selangor, Malaysia.

## Abstract

**Objective:**

Effective prevention and control measures are essential to contain outbreaks of infectious diseases, such as coronavirus disease (COVID-19). Understanding the characteristics of case clusters can contribute to determining which prevention and control measures are needed. This study describes the characteristics of COVID-19 case clusters in Malaysia, the method used to detect a cluster’s index case and the mode of early transmission, using the seven cluster categories applied in Malaysia.

**Methods:**

This cross-sectional study collected publicly available data on COVID-19 clusters occurring in Malaysia from 1 March 2020 to 31 May 2021. The characteristics of cases were described by category, and their associations with several outcomes were analysed. Descriptive analyses were performed to explore the method used to detect the index case and the mode of early transmission, according to cluster category.

**Results:**

A total of 2188 clusters were identified. The workplace cluster category had the largest proportion of clusters (51.5%, 1126/2188 clusters), while the custodial settings category had the largest median cluster size (178 cases per cluster) and longest median duration of cluster (51 days). The high-risk groups category had the highest mortality. There were significant differences in cluster size, duration and rate of detection across the categories. Targeted screening was most commonly used to detect index cases, especially in custodial settings, and in imported and workplace clusters. Household–social and social–workplace contacts were the most common modes of early transmission across most categories.

**Discussion:**

Targeted screening might effectively reduce the size and duration of COVID-19 clusters. Measures to prevent and control COVID-19 outbreaks should be continually adjusted based on ongoing assessments of the unique context of each cluster.

Coronavirus disease (COVID-19) was first detected in Malaysia on 25 January 2020, with the first COVID-19 cluster recorded approximately 1 month later, on 1 March 2020. (*1*, *2*) The Malaysian Ministry of Health defined a COVID-19 cluster as “a concentration of infections in the same area at the same time.” (*3*)

Identifying case clusters early in an outbreak is crucial because it allows health authorities to link cases to the same source, trace close contacts and isolate all identified cases (i.e. the clusters of cases stage). (*4*-*6*) When cases become widespread in a community and are not clearly linked to a source of infection (i.e. during community transmission) and when an increasing number of severe cases require hospitalization, the health-care system can become overburdened, and so its capacity to follow up on new clusters may be limited. (*6*) Thus, identifying clusters early and implementing containment measures to stop further transmission can limit the spread of an outbreak.

Categorizing clusters of COVID-19 cases and analysing their characteristics allows policy-makers to design targeted public health measures to control outbreaks in key areas and populations. (*7*) Each country has a different classification system for case clusters. For instance, a study from China classified clusters into combinations of the following categories: family, social, travel, work, community or vehicle. (*8*) In Malaysia, COVID-19 clusters are divided into seven categories: community, custodial settings, educational institutions, high-risk groups, imported, religious organizations and workplace, based on either the profile or the locality of the index case when the cluster was detected. (*3*, *5*) Clusters in different categories behave distinctively due to differences in context, setting and demographics and, therefore, different categories require different containment approaches. (*7*)

Several local studies in Malaysia (*4*, *9*, *10*) described the transmission and management of selected clusters of COVID-19 cases, but none has summarized the characteristics of all of the clusters. Understanding the characteristics of the different categories is critical to ensuring that policy-makers can tailor preventive measures – such as vaccination programmes, targeted screening, and health promotion and education programmes – to contain the clusters of cases stage. (*6*, *11*) Knowing the origin of a cluster and how the infection was transmitted facilitates the selection of mitigation measures. It also serves as a learning point to strengthen the health system to respond to future outbreaks.

Hence, this study aims to describe the characteristics, detection methods and modes of early transmission of COVID-19 cases using Malaysia’s seven categories of clusters. To our knowledge, this is the first study of COVID-19 clusters in Malaysia that attempts to summarize the methods used to detect the index case and modes of early transmission for different categories of clusters and explore the relationships between the characteristics of the clusters.

## Methods

### Sources of data

This cross-sectional study included clusters of COVID-19 cases in Malaysia that were publicly reported from 1 March 2020 to 31 May 2021. Detailed information during the earliest stages of the pandemic was published up until 31 May 2021, and this included the method used to detect the index case and the modes of early transmission. Subsequently, the public reporting format was changed as the number of cases increased. Data were collected from the following publicly available sources: COVID-19 data on GitHub, (*1*) the Ministry of Health COVID-19 web site (*12*) and the Ministry’s social media accounts (e.g. Facebook and Twitter), other government agencies and their official web sites, and local news portals (**Box 1**).

**Box 1:**
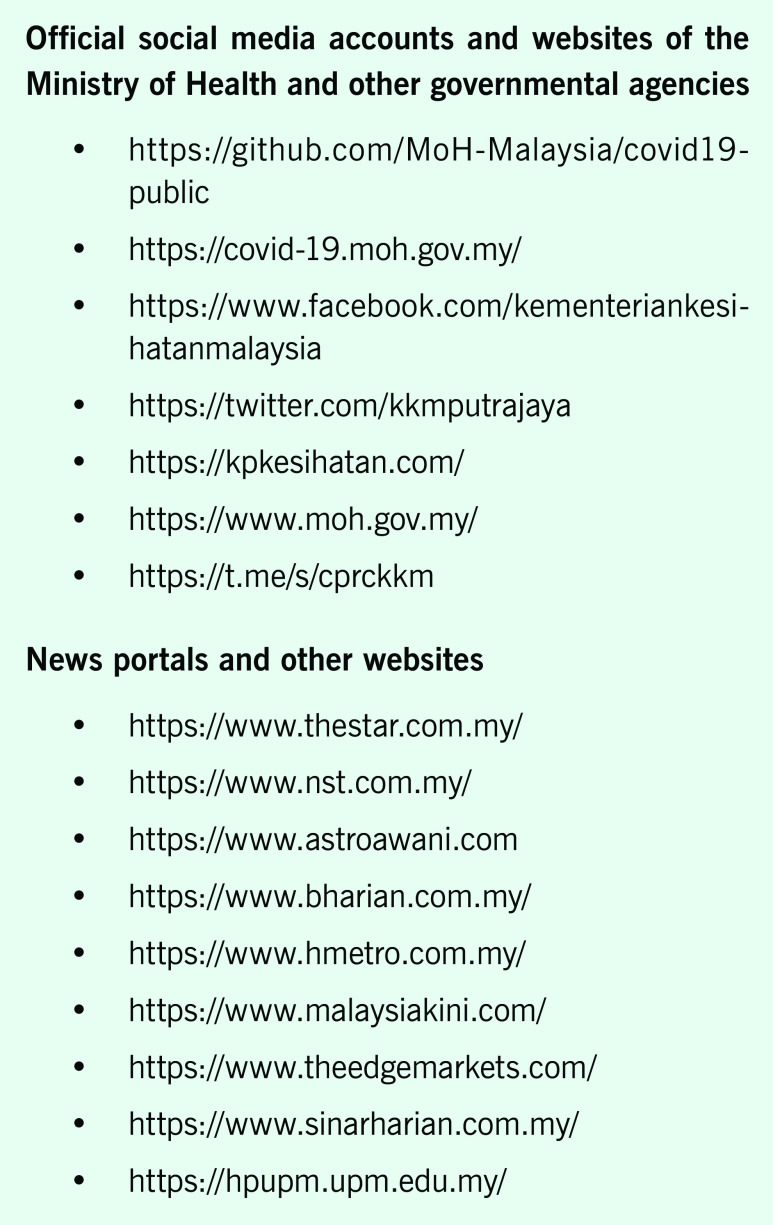
Sources of data on COVID-19 clusters reported in Malaysia, 1 March 2020–31 May 2021

[**insert** Box 1]

For every COVID-19 index case identified, the Ministry would perform contact tracing and epidemiological investigations before officially reporting the cluster to the public. (*4*) Clusters were declared to have ended after no new cases were detected (*9*) or the last person detected within the cluster had been asymptomatic for 28 consecutive days (i.e. double the incubation period of COVID-19). (*13*) If this information was unavailable, the authors deemed the cluster end date to be 28 days after the date of onset of the last symptom, as per the definition above.

The data collected included cluster size, duration, number of deaths, number of COVID-19 diagnostic tests performed, detection method and mode of transmission. The case–fatality rate and test positivity rate were then calculated. The test positivity rate was defined as a proportion: the total number of cases who tested positive for COVID-19 in a particular cluster divided by the total number of individuals screened for the particular cluster. Clusters were put into one of the seven categories described above. The four categories used to assess the detection method were: targeted screening, symptomatic screening, self-screening (i.e. screening voluntarily undertaken by individuals and organizations) and not reported (**Table 1**).

**Table 1:** Definitions of categories, detection methods and modes of transmission used for clusters of COVID-19 cases, Malaysia, 1 March 2020–31 May 2021

Variable	Definition
Cluster category (*3*)	Community: clusters originating from activities in the community, including at home, at large communal dwellings (i.e. longhouses), and during festivals, funerals, receptions and weddings Custodial setting: clusters originating in any custodial setting, including prisons, lock-ups and immigration detention depots Educational institution: clusters originating in Ministry of Education institutions, higher education institutions and educational institutions not affiliated with the Ministry of Education High-risk group: clusters originating among high-risk groups in aged-care facilities, government and private hospitals, nurseries, dialysis centres and welfare centres Imported: clusters in which the index case contracted COVID-19 in another country Religious organizations: clusters originating from religious activities Workplace: clusters originating in places of employment
Total no. of cases (i.e. cluster size)	The total number of people testing positive for COVID-19 who were linked to a particular cluster when it was reported to have ended
Duration	The number of days between the date on which a particular cluster was officially reported by the Ministry of Health and the date on which it was declared to have ended
Case–fatality rate (%)	The proportion of cases in a cluster who died from COVID-19 divided by the total number of COVID-19 cases in the cluster
Detection method	The method used to detect the index case for each cluster Targeted screening: refers to planned screening at points of entry; for contacts of cases; individuals applying for interstate or interdistrict travel permits within Malaysia when Movement Control Orders were in effect; workers at wet markets; health-care workers; patients before surgery and admission to hospital; during postmortem examinations; for individuals with influenza-like illness or severe acute respiratory infection; people in areas under an Enhanced Movement Control Order; staff and residents at aged-care facilities; staff and inmates in custodial facilities, including prisons, immigration detention centres, drug rehabilitation centres and other custodial settings; workers at construction sites; security guards; individuals in communities at risk of COVID-19, including those in close contact with COVID-19 cases; workers and staff at factories; staff and students at educational facilities; staff and customers at shopping malls and supermarkets; and employees at workplaces that did not fall under any other workplace screening mechanism in this list Symptomatic testing: refers to testing of individuals who have symptoms of COVID-19 Self-screening: refers to testing voluntarily performed by individuals or organizations Not reported: the detection method was not made publicly available
Mode of early transmission	Custodial setting: includes clusters spread within or from prisons, immigration detention centres, drug rehabilitation centres and other custodial settings; includes transmission among inmates and staff Educational institution: includes clusters spread within or from all educational institutions, such as primary, second and tertiary schools, preschools and nurseries; includes transmission among staff and students Household: refers to spread through household contacts who live under the same roof, including in workers’ accommodation, dormitories and hostels; this category excludes aged-care homes Social: includes transmission through gatherings at social, festive and cultural events, and through other types of community and residential areas, such as contacts among neighbours Workplace (general): includes transmission among local workers, foreign workers and in the place of employment Others: refers to modes of transmission that are not covered by the categories described above Not reported: refers to modes of early transmission that were not announced or not specified, such as a close contact

The mode of early transmission for a cluster was defined as the reported transmission mode for the index case or for earlier generations of cases that infected other cases within the cluster, beginning from the date the index case was detected until the official start date of the cluster. The category assigned by the research team was based on descriptions and illustrations of clusters provided by the Ministry of Health. The mode of early transmission could be a single mode or a combination of modes. For example, the household–social category indicated that cases were spread through household and social contacts.

### Analyses

The characteristics of each cluster were assessed and the cluster was assigned to one of the seven categories. Whether the data fit a normal distribution was explored using histograms and acceptable skewness and kurtosis values of between −2 and +2. (*14*) The characteristics were summarized using frequencies and the percentage of occurrence for categorical data, and using medians and interquartile ranges (IQRs) for continuous data. We also described the detection methods and modes of early transmission among COVID-19 clusters using the categories.

The differences between the seven categories (i.e. total cases/cluster size, duration and test positivity rate) were analysed using the Kruskal–Wallis test and, subsequently, Dunn’s test because the continuous data were not normally distributed. The level of significance was *P* < 0.05. All analyses were performed using R software (version 4.2.1, R Core Team, Vienna, Austria) and Microsoft Excel (2019).

## Results

### Description of COVID-19 clusters

From 1 March 2020 until 31 May 2021, there were 2188 COVID-19 clusters reported in Malaysia, comprising 243 377 cases. About half of the clusters (*n* = 1126, 51.5%), comprising 145 018 cases, originated in a workplace, and one quarter (*n* = 548, 25.0%), comprising 37 105 cases, occurred in the community (**Table 2**).

**Table 2:** Characteristics of clusters of COVID-19 cases, Malaysia, 1 March 2020–31 May 2021 (*n* = 2188)

Cluster category	No. (%) of clusters	Total no. (%) of COVID-19 cases	Total no. (%) of deaths	Median no. (IQR) of cases per cluster^a^	Median no. (IQR) of days duration^b^	Median% (IQR) test positivity rate^c^
Workplace	1126 (51.5)	145 018 (59.6)	121 (0.08)	44 (78)	39 (17)	25.0 (28.2)
Community	548 (25.0)	37 105 (15.2)	213 (0.6)	33 (48)	39 (14)	19.9 (28.6)
Educational institution	184 (8.4)	12 722 (5.2)	17 (0.13)	35.5 (55.3)	39 (13.3)	17.3 (22.8)
Religious organization	136 (6.2)	15 342 (6.3)	146 (0.95)	54 (92)	41 (16)	24.5 (27.2)
High-risk group	103 (4.7)	3858 (1.6)	108 (2.8)	26 (26.5)	37 (15)	21.8 (40.0)
Custodial setting	62 (2.8)	27 232 (11.2)	23 (0.08)	178 (410)	51 (45.5)	30.3 (33.3)
Imported	29 (1.3)	2100 (0.9)	13 (0.6)	8 (42)	33 (13)	17.1 (25.5)
Total no. of clusters	2188 (100)	243 377 (100)	641 (0.3)	39 (68)	39 (16)	23.0 (29.1)

IQR: interquartile range.

**^a^** *H* statistic for cluster size = 116.85, df = 6, *P* < 0.001.

^b^ *H* statistic for cluster duration = 38.71, df = 6, *P* < 0.001.

^c^ *H* statistic for positivity rate = 51.08, df = 6, *P* < 0.001.

The clusters with the largest median size were those in custodial settings (median: 178 cases; IQR: 410 cases), despite these comprising only 2.8% (62/2188) of the reported clusters. Cluster size was associated with cluster category (*P* < 0.001), with statistically significant differences in the median cluster size between all pairs of categories, except for community–educational institution, community–high-risk group, high-risk group–imported and religious organization–workplace (**Table 3**). Thus, clusters in custodial settings and religious organizations were significantly larger than those in the other categories, while clusters from imported cases were significantly smaller than in other categories.

**Table 3:** Results from the post-hoc analysis using Dunn’s test for comparisons between cluster category and size, duration and positivity rate, Malaysia, 1 March 2020–31 May 2021

Cluster category pair	Cluster size vs cluster category		Cluster duration vs cluster category		Positivity rate vs cluster category	
	*Z*	Adjusted *P*	*Z*	Adjusted *P*	*Z*	Adjusted *P*
Community–custodial setting	−7.608	< 0.001	−4.996	0.000	−3.478	0.003
Community–educational institution	−1.448	0.155	−0.174	0.862	1.446	0.194
Community–high-risk group	1.961	0.058	1.161	0.304	−0.897	0.431
Community–imported	3.106	0.003	2.551	0.025	1.153	0.308
Community–religious organization	−4.315	< 0.001	−1.774	0.133	−2.012	0.093
Community–workplace	−5.691	< 0.001	−0.557	0.638	−5.045	< 0.001
Custodial–educational institution	6.102	< 0.001	4.458	< 0.001	4.013	< 0.001
Custodial setting–high- risk group	7.652	< 0.001	4.940	< 0.001	2.300	0.056
Custodial setting–imported	7.162	< 0.001	5.136	< 0.001	3.048	0.010
Custodial setting–religious organization	3.955	< 0.001	3.259	0.004	1.783	0.130
Custodial setting–workplace	5.542	< 0.001	4.909	< 0.001	1.559	0.179
Educational institution–high-risk group	2.714	0.009	1.133	0.300	−1.784	0.142
Educational institution–imported	3.580	0.001	2.507	0.026	0.483	0.629
Educational institution–religious organization	−2.565	0.014	−1.372	0.223	−2.795	0.018
Educational institution–workplace	−2.176	0.036	−0.179	0.901	−4.854	< 0.001
High-risk group–imported	1.813	0.077	1.719	0.138	1.503	0.186
High-risk group–religious organization	−4.778	< 0.001	−2.256	0.046	−0.739	0.483
High-risk group–workplace	−4.926	< 0.001	−1.493	0.190	−1.617	0.171
Imported–religious organization	−4.915	< 0.001	−3.207	0.004	−2.017	0.102
Imported–workplace	−4.723	< 0.001	−2.739	0.016	−2.565	0.031
Religious organization–workplace	1.288	0.198	1.553	0.181	−0.771	0.487

Clusters in custodial settings had the longest median duration (median: 51 days; IQR: 45.5 days), while imported clusters had the shortest duration (median: 33 days; IQR: 13 days) (**Table 2**). The duration of clusters was significantly different between categories (*P* < 0.001), with the duration of clusters in custodial settings being significantly longer than in all other categories in the paired analysis. In contrast, the duration of imported clusters was significantly shorter than that in all other categories (**Table 2**).

The test positivity rate was highest for clusters in custodial settings (median: 30.3%; IQR: 33.3%), while the lowest test positivity rates were in imported clusters (median: 17.1%; IQR: 25.5%) and clusters in educational institutions (median: 17.3%; IQR: 22.8%). The test positivity rate was significantly different between categories (*P* < 0.001), with statistically significant differences in median test positivity rates for the following pairs: custodial setting–community, custodial setting–educational institution, custodial setting–imported, educational institution–religious organization, educational institution–workplace, community–workplace and imported–workplace (**Table 3**).

There were 641 deaths, with an average case–fatality rate per cluster of 0.26% (**Table 2**). High-risk groups had the highest case–fatality rate (2.8%), but the majority of clusters (*n* = 1881, 86%) had no deaths.

### Detection methods

**Fig. 1:**
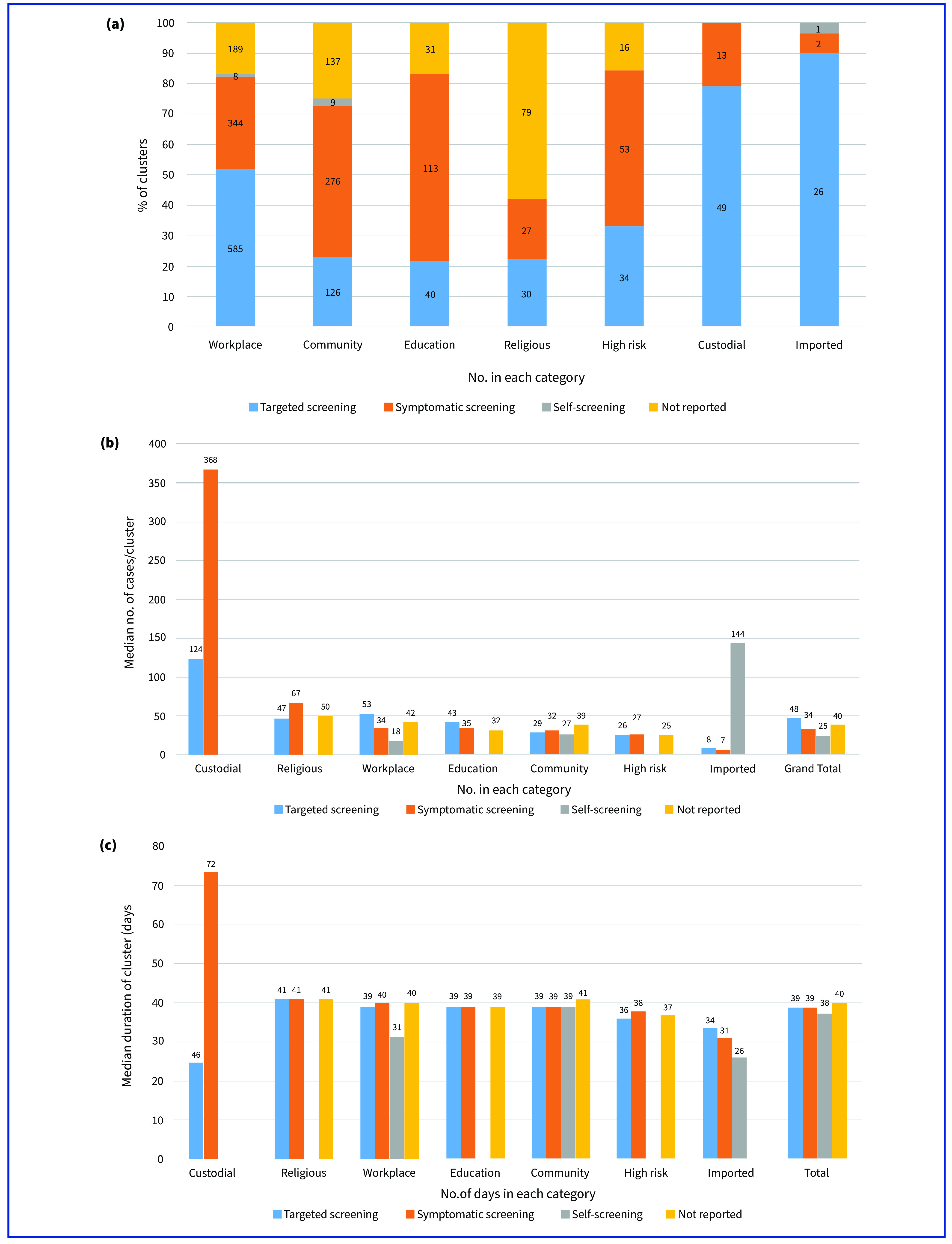
(a) Proportion of clusters of COVID-19, by method used to detect the index case and category; (b) median number of COVID-19 cases per cluster (cluster size), by detection method for the index case and category; (c) median duration of cluster, by detection method for the index case and category

Targeted screening detected 40.7% (*n* = 890) of all clusters, and it detected 79.0% (49/62) of clusters in custodial settings, 89.7% (26/29) of clusters among imported cases and 51.9% (585/1126) in workplaces. In contrast, more than half of clusters in educational institutions, the community and high-risk groups were detected through screening of individuals who were symptomatic (**Fig. 1a**).

Among the clusters in custodial settings, the largest median number of cases was 368, identified through symptomatic screening, which was threefold higher than for targeted screening (124 cases) (**Fig. 1b**). The median numbers of cases in other categories were similar across the different detection methods. Similarly, clusters in custodial settings, where the index case was detected through symptomatic screening, had a median duration of 72 days, 57% longer than for clusters detected using targeted screening (46 days). The duration for other categories was similar (approximately 40 days) (**Fig. 1c**).

[**insert** Figure 1]

### Mode of transmission

The most frequent modes of transmission were through household–social, workplace and social contacts, contributing to approximately two thirds of all COVID-19 clusters in Malaysia (**Table 4**). The transmission mode for most clusters in custodial settings was within the setting (59.7%, 37/62), with 20.9% (13/62) of cases transmitted through social interactions. About 45.1% (508/1126) of workplace clusters were transmitted within workplaces, with another 15.4% (173/1126) and 16.7% (188/1126) transmitted through household–social and social contacts, respectively. Furthermore, between 32% and 75% of clusters in the community, educational institutions, high-risk groups, religious organizations and the workplace were transmitted through household–social and social contacts (**Table 4**).

**Table 4:** Number and proportion of COVID-19 clusters, by category and mode of transmission, Malaysia, 1 March 2020–31 May 2021

Cluster category	No. (%) of clusters by mode of transmission^a^									
	Household–social	Workplace	Social	Workplace–household–social	Educational institution–household–social	Household–workplace	Custodial	Other	Not reported	Total
Workplace	173 (15.4)	508 (45.1)	188 (16.7)	60 (5.3)	0	42 (3.7)	1 (0.1)	14 (1.2)	140 (12.4)	1126 (100)
Community	321 (58.6)	2 (0.4)	90 (16.4)	7 (1.3)	2 (0.4)	3 (0.5)	0	5 (0.9)	118 (21.5)	548 (100)
Educational institution	42 (22.8)	2 (1.1)	46 (25.0)	2 (1.1)	72 (39.1)	2 (1.1)	0	4 (2.2)	14 (7.6)	184 (100)
Religious organization	50 (36.8)	1 (0.7)	14 (10.3)	4 (2.9)	1 (0.7)	0	0	11 (8.1)	55 (40.4)	136 (100)
High-risk group	33 (32.0)	0	39 (37.9)	0	0	3 (2.9)	0	17 (16.5)	11 (10.7)	103 (100)
Custodial setting	2 (3.2)	0	13 (21.0)	0	0	0	37 (59.7)	5 (8.1)	5 (8.1)	62 (100)
Imported	1 (3.4)	7 (24.1)	2 (6.9)	2 (6.9)	0	2 (6.9)	1 (3.4)	9 (31.0)	5 (17.2)	29 (100)
Total	622 (28.4)	520 (23.8)	392 (17.9)	75 (3.4)	75 (3.4)	52 (2.4)	39 (1.8)	65 (3.0)	348 (15.9)	2188 (100)

^a^ All transmission modes assigned to a cluster were mutually exclusive and independent of any other mode.

## Discussion

In this study of COVID-19 in Malaysia reported from 1 March 2020 to 31 May 2021, the largest number of clusters occurred in the workplace, while custodial settings had the largest median cluster size and longest median duration. The highest mortality rate was in the high-risk groups. Targeted screening was the most frequently used detection method for clusters, especially for custodial settings, among imported cases and for workplace clusters. The most common modes of early transmission across all categories were through household–social, social and workplace contacts, except for the custodial setting category, where transmission primarily occurred through contact among prisoners.

Workplace clusters contributed the largest number of cluster cases in Malaysia, accounting for 51.5% of these cases. This suggests that ensuring physical distancing and well ventilated workplaces are essential to prevent transmission in this setting. (*15*) In Malaysia, overcrowded living and working environments for foreign workers were reported to be one contributor to high transmission in the workplace at the beginning of the pandemic. (*9*, *16*, *17*) To mitigate the situation, the Malaysia Workers’ Minimum Standards of Housing and Amenities Act 1990 was amended in 2020 to improve the living conditions of workers, and employers and those who provide their accommodation can face maximum fines of 50 000 Malaysian ringgit (US$ 11 331) for not meeting the criteria. (*2*, *18*) For other workplaces, strict standard operating procedures were enforced to prevent transmission at work, and these required physical distancing, sanitizing the premises and restricting the maximum number of clients and workers within an office. (*19*)

The clusters in Malaysia had a higher median number of cases compared with clusters in the Republic of Korea (39 cases versus 27 cases, respectively). (*8*, *17*) This could be due to the use of different definitions of clusters: the Republic of Korea defined a COVID-19 cluster as a group of more than five cases that had the same point of contact, such as a location or an event, and excluded cases with secondary epidemiological links, such as transmission occurring within the same household; (*8*) Malaysia defined a cluster as a concentration of infections occurring in the same area at the same time. (*3*, *10*) Moreover, in Malaysia, COVID-19 cases within each cluster, particularly those beyond first-generation transmission, were not limited to occurring in the same setting as the index case, which could explain the larger size of clusters in Malaysia.

Although not many clusters occurred in custodial settings, these settings had the highest median number of cases per cluster and the longest duration. This may be due to the living conditions in custodial settings, such as prisons and detention centres, where the implementation of public health interventions – including physical distancing, mask-wearing and disinfection – was limited. (*11*) Additionally, Malaysian prisons are 13–36% over their designated capacity, (*3*, *20*, *21*) and local studies have shown that COVID-19 spreads easily in densely populated and confined spaces. (*22*, *23*) Yet the restricted movement of inmates in custodial settings eased contact tracing and screening efforts for suspected cases, so fewer resources were required to complete these tasks compared with other settings. Since the source of infection for most inmates could be identified, and clusters of inmates who tested positive had relatively more people and a longer duration of spread, clusters in custodial settings were the largest and had the longest duration compared with other cluster categories. The isolated conditions in custodial settings may also explain the relatively higher test positivity rate among clusters in these settings, as all suspected cases within the settings were screened. (*11*) Malaysia implemented several mitigation measures to reduce and contain the spread of COVID-19 within custodial settings, including setting up temporary detention centres, treatment centres in prisons and makeshift hospitals. (*24*) All new inmates were screened and isolated, if necessary, before being transferred to a permanent cell. (*25*)

Clusters in high-risk groups – which included those in health-care facilities, long-term care facilities and early childhood education and care settings (*3*) – had the highest case–fatality rate, at 2.8%. Other studies have shown that mortality was higher for residents in long-term care facilities (*26*, *27*) and for hospitalized patients (*28*) compared with other populations in the community. This is because comorbidities increase the risk of complications and death. (*11*)

The analysis of detection methods showed that targeted screening was the most common detection method for custodial settings, and imported and workplace clusters. Symptomatic screening was the predominant method used for detecting cases in the community, in educational institutions and among high-risk groups. This suggests that a targeted screening method could be more effective when public health authorities have more information about individuals’ identities and movements. However, the situation differed for clusters among high-risk groups, which had higher case–fatality rates, with more than half (52%) of index cases detected through symptomatic rather than targeted screening (33% detected). In addition to causing excess deaths in long-term care and health-care facilities, COVID-19 outbreaks in early childhood education and care settings have disrupted children’s learning and development, as well as carers’ routines. (*29*) Therefore, high-risk groups need both targeted and symptomatic screening to limit the spread of COVID-19 and reduce mortality. (*30*)

Our results indicate that early transmission in the community occurs mostly through household and social contacts, in educational institutions, among high-risk groups, through religious organizations and in workplace settings. These observations are supported by a meta-analysis by Lei et al. (*31*) that found the risk of household secondary attack rate for COVID-19 (i.e. the risk of transmission from an index case to an exposed contact) is approximately 10 times greater than the risk from other contacts. This is because strategies such as physical distancing, quarantine and mask-wearing, which are effective in normal settings, might not work well within a household due to crowded living spaces and behavioural factors. (*32*) Similarly, two local online surveys in Malaysia in April and July 2020 about health and social behaviours showed that approximately 50–60% of respondents were still meeting in person and socializing with friends and relatives during the Movement Control Order, which was put in place to slow the spread of COVID-19. (*33*, *34*) Findings from these studies might explain why household–social and social transmission were the primary modes of early transmission in the community, educational institutions, religious organizations, workplace settings and among high-risk groups.

Our study also found that about 45% of the transmission that occurred among work colleagues was limited to the workplace. To address this, Malaysia implemented several regulations to control outbreaks in the workplace during the pandemic. (*35*, *36*) Indeed, a literature review by Lynch et al. (*37*) found that preventive measures effectively lowered the transmission rate of COVID-19 in workplaces. Nevertheless, as workers interact with other individuals within their household and community, (*37*) COVID-19 could be spread. This explains how 41% of workplace clusters spread through household and social contacts during the early stage of the cluster.

This study analysed all case clusters in Malaysia during the period for which data were publicly available. Although the study included a large amount of aggregated data from multiple platforms, it has some limitations. The data did not include all details about each individual case in each cluster, such as information about vaccination status or variants of severe acute respiratory syndrome coronavirus 2. Therefore, the study was unable to evaluate the dominant variants in the community or the effect of vaccination on the transmission of cases within clusters. The Malaysian vaccination programme was initiated in February 2021, and the vaccination rate was 3.35% as of 31 May 2021. (*1*) Moreover, due to the cross-sectional nature of the study, the transmission dynamics of COVID-19 were not captured, and this may have affected the results. Future studies using more complete data are required to explore these areas.

Although each mode of early transmission assigned to a cluster was mutually exclusive and independent of the others, when an individual was exposed to multiple clusters concurrently, they had multiple possibilities for their source of infection, making contact tracing challenging. As such, the decision about the mode of early transmission and assignment to a cluster by case investigators was based on the most likely source of infection for individuals. Lastly, due to the large number of clusters (*n* = 2188), slight differences in inferential tests may contribute to statistically significant differences. Therefore, our findings should be interpreted with caution. (*38*)

In conclusion, the different categories of COVID-19 clusters reported in Malaysia from 1 March 2020 to 31 May 2021 had different characteristics and these were related to the context and setting of each category. Therefore, tailored strategies are needed to contain the spread of cases and depend on the category. Targeted screening might effectively reduce the size and duration of clusters. Prevention and control measures used against COVID-19 should be continually adjusted based on ongoing assessments of the unique context of each cluster category.

## References

[1] Ministry of Health Malaysia. official data on the COVID-19 epidemic in Malaysia. Putrajaya: Ministry of Health Malaysia; 2022. Available from: https://github.com/MoH-Malaysia/covid19-public, accessed 9 January 2023.

[2] Ang ZY, Cheah KY, Shakirah MS, Fun WH, Anis-Syakira J, Kong YL, et al. Malaysia’s health systems response to COVID-19. Int J Environ Res Public Health. 2021 Oct 22;18(21):11109. 10.3390/ijerph182111109 34769629 PMC8583455

[3] COVID-19 technical report 2020–2021: the challenging years. COVID-19 clusters. Setia Alam: National Institutes of Health, Malaysia; 2022. Available from: https://heyzine.com/flip-book/b8c7a075af.html#page/119, accessed 13 February 2023.

[4] Hashim JH, Adman MA, Hashim Z, Mohd Radi MF, Kwan SC. COVID-19 epidemic in Malaysia: epidemic progression, challenges, and response. Front Public Health. 2021 May 7;9:560592. 10.3389/fpubh.2021.560592 34026696 PMC8138565

[5] Malaysia: coronavirus disease (COVID-19) situation report: weekly report for the week ending 27 June 2021. Cyberjaya: WHO Representative Office for Malaysia, Brunei Darussalam and Singapore; 2021. Available from: https://www.who.int/malaysia/internal-publications-detail/covid-19-in-malaysia-situation-report-49, accessed 14 February 2023.

[6] Considerations for implementing and adjusting public health and social measures in the context of COVID-19: interim guidance, 14 June 2021. Geneva: World Health Organization; 2021. Available from: https://apps.who.int/iris/rest/bitstreams/1351572/retrieve, accessed 28 February 2023.

[7] Choi YJ, Park MJ, Park SJ, Hong D, Lee S, Lee KS, et al. Types of COVID-19 clusters and their relationship with social distancing in the Seoul metropolitan area, South Korea. Int J Infect Dis. 2021 May;106:363–9. 10.1016/j.ijid.2021.02.058 33609772 PMC7889017

[8] Xiao S, Liu Y, Liu F, Zhang H, Zhang F, Wang L. Epidemiological characteristics of COVID-19 clusters in Hainan, China. Medicine (Baltimore). 2021 Oct 22;100(42):e27512. 10.1097/MD.0000000000027512 34678885 PMC8542154

[9] Danial M, Arulappen AL, Ch’ng ASH, Looi I. Mitigation of COVID-19 clusters in Malaysia. J Glob Health. 2020 Dec;10(2):0203105. 10.7189/jogh.10.0203105 33403108 PMC7750020

[10] Safuan S, Edinur HA. Sri Petaling COVID-19 cluster in Malaysia: challenges and the mitigation strategies. Acta Biomed. 2020 Nov 10;91(4):e2020154. 10.23750/abm.v91i4.10345 33525245 PMC7927555

[11] Mansor Z, Mokhtar SA. COVID-19 clusters in Malaysia: a descriptive analysis. Adv Health Sci Res. 2022;44:5–11. 10.2991/ahsr.k.220108.002

[12] COVID-19 Malaysia. Putrajaya: Ministry of Health, Malaysia; 2023 (in Malaysian). Available from: https://covid-19.moh.gov.my/, accessed 1 March 2021.

[13] COVID-19. MOH declares end of 13 clusters. Kuala Lumpur: Astro Awani; 2020. Available from: https://www.astroawani.com/berita-malaysia/covid19-moh-declares-end-13-clusters-243866, accessed 14 June 2023.

[14] George DB, Mallery P. SPSS for Windows step by step: a simple study guide and reference, 17.0 update. 10th ed. Boston (MA): Pearson; 2010.

[15] COVID-19 transmission and protective measures: last updated 2022. Manila: WHO Regional Office for the Western Pacific; 2022. Available from: https://www.who.int/westernpacific/emergencies/covid-19/information/transmission-protective-measures, accessed 8 March 2023.

[16] Mohamed Radhia NA, Basyir M. Surge in Covid-19 clusters at Klang Valley workplaces. Kuala Lumpur: New Straits Times; 2020. [cited 2023 Jun 14]. Available from: Available from https://www.nst.com.my/news/nation/2020/11/641918/surge-covid-19-clusters-klang-valley-workplaces

[17] Spaccaferri G, Calba C, Vilain P, Garras L, Durand C, Pilorget C, et al.; Regional MONIC group. COVID-19 hotspots through clusters analysis in France (may-October 2020): where should we track the virus to mitigate the spread? BMC Public Health. 2021 Oct 11;21(1):1834. 10.1186/s12889-021-11857-8 34635085 PMC8503705

[18] Lim I. New rules for employees’ minimum housing standards from Sept 1: employers to comply or be fined RM50,000. Kuala Lumpur: Malay Mail; 2020. [cited 2023 Mar 8]. Available from: Available from https://www.malaymail.com/news/malaysia/2020/08/30/new-rules-for-employees-minimum-housing-standards-from-sept-1-employers-to/1898538

[19] Arahan Ketua Pengarah – Peraturan 11 P.U.(A) 136/2020: prosedur operasi standard pembukaan semula ekonomi [The Order of the Director-General – Regulation 11 Legal Notification (A) 136/2020: standard operating procedures for reopening of economy]. Putrajaya: Ministry of Health, Malaysia; 2020 (in Malaysian). Available from: https://covid-19.moh.gov.my/faqsop/sop-pembukaan-ekonomi-kemaskini-03-jun-2020/SOP-All-PKPB-.pdf, accessed 2 January 2021.

[20] Chung N. Our prisons are overcrowded, says deputy minister. Selangor: Free Malaysia Today; 2021. [cited 2023 Mar 8]. Available from: Available from https://www.freemalaysiatoday.com/category/nation/2021/09/28/our-prisons-are-overcrowded-says-deputy-minister/

[21] Bunyan J. Prisons Dept reveals jail congestion at 36pc, taking measures to reduce overcrowding. Kuala Lumpur: Malay Mail; 2023. [cited 2023 Mar 8]. Available from: Available from https://www.malaymail.com/news/malaysia/2023/02/03/prisons-dept-reveals-jail-congestion-at-36pc-taking-measures-to-reduce-overcrowding/53253

[22] Aw SB, Teh BT, Ling GHT, Leng PC, Chan WH, Ahmad MH. The COVID-19 pandemic situation in Malaysia: lessons learned from the perspective of population density. Int J Environ Res Public Health. 2021 Jun 18;18(12):6566. 10.3390/ijerph18126566 34207205 PMC8296337

[23] Md Iderus NH, Lakha Singh SS, Mohd Ghazali S, Yoon Ling C, Cia Vei T, Md Zamri ASS, et al. Correlation between population density and COVID-19 cases during the third wave in Malaysia: effect of the Delta variant. Int J Environ Res Public Health. 2022 Jun 17;19(12):7439. 10.3390/ijerph19127439 35742687 PMC9223655

[24] Technical report 2022: Kedah State Health Department. Alor Setar: Kedah State Health Department; 2022. Available from: https://jknkedah.moh.gov.my/images/penerbitan/LAPORAN_TEKNIKAL_JKN_KEDAH_2022.pdf, accessed 5 January 2023.

[25] Annex 27a: Tindakan Pencegahan, Kawalan Infeksi dan Penggunaan Personal Protective Equipment (PPE), Di Fasiliti Tahanan dan Rumah Perlindungan [Annex 27a: Infection prevention and control, and usage of personal protective equipment (PPE) at detention facilities and shelter homes]. Putrajaya: Ministry of Health, Malaysia; 2021 (in Malaysian). Available from: https://covid-19.moh.gov.my/garis-panduan/garis-panduan-kkm/ANNEX_27a_SOP_for_Depot_Tahanan_Imigresen_Penjara_dan_Lock_Up_24092021.pdf, accessed 14 July 2023.

[26] Ballin M, Bergman J, Kivipelto M, Nordström A, Nordström P. Excess mortality after COVID-19 in Swedish long-term care facilities. J Am Med Dir Assoc. 2021 Aug;22(8):1574–1580.e8. 10.1016/j.jamda.2021.06.010 34174196 PMC8223135

[27] Betini R, Milicic S, Lawand C. The impact of the COVID-19 pandemic on long-term care in Canada. Healthc Q. 2021 Oct;24(3):13–5. 10.12927/hcq.2021.26625 34792442

[28] Ponsford MJ, Ward TJC, Stoneham SM, Dallimore CM, Sham D, Osman K, et al. A systematic review and meta-analysis of inpatient mortality associated with nosocomial and community COVID-19 exposes the vulnerability of immunosuppressed adults. Front Immunol. 2021 Oct 6;12:744696. 10.3389/fimmu.2021.744696 34691049 PMC8526940

[29] La Valle I, Lewis J, Crawford C, Paull G, Lloyd E, Ott E, et al. Implications of COVID for early childhood education and care in England. London: Centre for Evidence and Implementation; 2022. [cited 2023 Jan 23]. Available from: Available from https://www.familyandchildcaretrust.org/sites/default/files/Resource%20Library/Implications%20of%20Covid%20for%20ECEC%20in%20England%20-%20June%202022_0.pdf

[30] Gao Z, Xu Y, Sun C, Wang X, Guo Y, Qiu S, et al. A systematic review of asymptomatic infections with COVID-19. J Microbiol Immunol Infect. 2021 Feb;54(1):12–6. 10.1016/j.jmii.2020.05.001 32425996 PMC7227597

[31] Lei H, Xu X, Xiao S, Wu X, Shu Y. Household transmission of COVID-19-a systematic review and meta-analysis. J Infect. 2020 Dec;81(6):979–97. 10.1016/j.jinf.2020.08.033 32858069 PMC7446647

[32] Cerami C, Popkin-Hall ZR, Rapp T, Tompkins K, Zhang H, Muller MS, et al. Household transmission of severe acute respiratory syndrome coronavirus 2 in the United States: living density, viral load, and disproportionate impact on communities of color. Clin Infect Dis. 2022 May 30;74(10):1776–85. 10.1093/cid/ciab701 34383889 PMC8436395

[33] Perialathan K, Ahmad M, Yong TSM, Johari MZ, Jaafar N, Juatan N, et al. Online survey on public’s understanding, attitude and practice related to physical distancing. In: Zainudin A, Abdul Kadir K, Suhaimi SA, editors. An insight of behaviour research during COVID-19 in Malaysia. Setia Alam: Institute for Health Behavioural Research; 2020:23–5. Available from: https://iptk.moh.gov.my/images/insight_book/IPTKBook.pdf, accessed 2 January 2023.

[34] Ithnain N, Panting AJ, Kassim R, Amirudin N, Suhaimi SA, Musa KA, et al. Health & social behaviour during movement control order (MCO) following COVID-19: an online survey among adult internet users in Malaysia. Key findings. Setia Alam: Institute for Health Behavioural Research; 2020. Available from: https://iptk.moh.gov.my/images/research/2020/HBMCO_INFOGRAFIK_IPTK_2020.pdf, accessed 2 January 2023.

[35] Arkib SOP. [Archive for SOPs]. Putrajaya: National Security Council, Government of Malaysia; 2020 (in Malaysian). Available from: https://www.mkn.gov.my/web/ms/arkib-sop/, accessed 15 July 2021.

[36] Soalan lazim (FAQ’s) berkaitan Perintah Kawalan Pergerakan Kementerian Perdagangan Antarabangsa dan Industri [Frequently asked questions (FAQs) related to the Movement Control Order of the Ministry of International Trade and Industry]. Putrajaya: Ministry of International Trade and Industry Malaysia; 2020 (in Malaysian). Available from: https://covid-19.moh.gov.my/faqsop/faq-umum/13.FAQ%20MITI_19_Mac_2020.pdf, accessed 13 May 2020.

[37] Lynch HN, Beckett EM, Dobyns LL, Thompson WJ, Divis HR, Encina E, et al. Literature review of the relative importance of household, community and social, and workplace settings on the probability of COVID-19 infection. J Public Health Emerg. 2022;6:35. 10.21037/jphe-22-12

[38] Faber J, Fonseca LM. How sample size influences research outcomes. Dental Press J Orthod. 2014 Jul-Aug;19(4):27–9. 10.1590/2176-9451.19.4.027-029.ebo 25279518 PMC4296634

